# Association Between the Decline in Pneumococcal Disease in Unimmunized Adults and Vaccine-Derived Protection Against Colonization in Toddlers and Preschool-Aged Children

**DOI:** 10.1093/aje/kwy219

**Published:** 2018-11-20

**Authors:** Daniel M Weinberger, Virginia E Pitzer, Gili Regev-Yochay, Noga Givon-Lavi, Ron Dagan

**Affiliations:** 1 Department of Epidemiology of Microbial Diseases, Yale School of Public Health, New Haven, Connecticut; 2 Infection Prevention and Control Unit, Sheba Medical Center, Ramat Gan, Israel; 3 Sackler School of Medicine, Tel Aviv University, Tel Aviv, Israel; 4 Pediatric Infectious Diseases Unit, Soroka University Medical Center, Be’er Sheva, Israel; 5 Faculty of Health Sciences, Ben-Gurion University of the Negev, Be’er Sheva, Israel

**Keywords:** herd immunity, indirect protection, invasive pneumococcal disease, pneumococcal carriage, pneumococcal conjugate vaccines, vaccination

## Abstract

Vaccinating children with pneumococcal conjugate vaccine (PCV) disrupts transmission, reducing disease rates in unvaccinated adults. When considering changes in vaccine dosing strategies (e.g., removing doses), it is critical to understand which groups of children contribute most to transmission to adults. We used data from Israel (2009–2016) to evaluate how the buildup of vaccine-associated immunity in children was associated with declines in invasive pneumococcal disease (IPD) due to vaccine-targeted serotypes in unimmunized adults. Data on vaccine uptake and prevalence of colonization with PCV-targeted serotypes were obtained from children visiting an emergency department in southern Israel and from surveys of colonization from central Israel. Data on IPD in adults were obtained from a nationwide surveillance study carried out in Israel. We compared the trajectory of decline of IPD due to PCV-targeted serotypes in adults with the decline of colonization prevalence and increase in vaccine-derived protection against pneumococcal carriage among different age groupings of children. The declines in IPD in adults were most closely associated with the declines in colonization and increased vaccination coverage among children in the age range of 36–59 months. This suggests that preschool-aged children, rather than infants, are responsible for maintaining the indirect benefits of PCVs.

Pneumococcal conjugate vaccines (PCVs) have had a well-documented impact on the incidence of invasive pneumococcal disease (IPD) and pneumonia in young children (1–3). PCVs reduce the burden of disease in 2 ways: They directly protect vaccinated persons who are exposed to the bacterium against invasive infections, and they indirectly protect vaccinated and unvaccinated persons (including adults) by reducing the prevalence of carriage of vaccine-targeted serotypes and thus reducing transmission. Colonization of the nasopharynx of young children represents the main reservoir for transmission of pneumococcus, and PCVs reduce the proportion of children who are colonized with serotypes targeted by the vaccine (4). The reduction in the burden of disease in unvaccinated adult age groups resulting from this indirect protection greatly outweighs the reduction in the burden of disease seen in vaccinated children alone (5).

While PCV programs have effectively reduced the burden of disease in many countries, the cost of the vaccine remains a major concern in both affluent and resource-poor settings. This issue has taken on particular urgency as some lower-income countries “graduate” from being eligible for financial support from the Global Alliance for Vaccines and Immunization (6).

To reduce costs while maintaining widespread use of the vaccine, there has been interest in reducing the number of doses of PCV delivered to children (7–9). Such a strategy could be used in populations that have already achieved strong reductions in disease because of the vaccine. Currently, most countries use either a 3 + 0 schedule (3 doses in the first 6 months of life, no booster dose) or a 2 + 1 schedule (2 doses in the first 6 months of life, 1 booster dose administered at 9–15 months of age). The newly proposed schedule would include just 1 primary dose and 1 booster dose (1 + 1). This schedule is being considered in the United Kingdom and is being evaluated by the World Health Organization. Reduced-dose schedules have been shown to be immunogenic (8, 10, 11). However, because of the importance of indirect protection that results from the use of PCVs, it would be desirable for any new dosing strategy to be able to maintain indirect protection (7).

To consider this issue, it is critical to determine which groups of young children contribute most to the indirect benefit of the vaccine for unimmunized adults. Epidemiologic and modeling studies of transmission focused on households and day-care centers suggest that toddlers and older children, rather than infants, drive transmission in the population (12–15). Likewise, the decline in IPD due to vaccine-targeted serotypes in adults is delayed and slower than the decline observed in vaccinated children (16). This might indicate that the transmission benefit (i.e., indirect effect) of vaccinating children during the first year of life is not realized until a few years later when those children reach an older age group.

In this study, we evaluated how the buildup of vaccine-associated immunity against colonization in different age categories was associated with declines in IPD due to vaccine-targeted serotypes in unimmunized age groups. To evaluate these associations, we used a unique and ongoing survey of children in Israel that allowed us to quantify vaccine uptake and IPD rates over time.

## METHODS

### Data sources

Seven-valent pneumococcal conjugate vaccine 7 (PCV7) was introduced into the national immunization program in Israel for all children in July 2009, with a catch-up campaign for all children under 24 months of age, and it was gradually replaced with 13-valent pneumococcal conjugate vaccine (PCV13) starting in November 2010. Carriage and vaccine uptake data for children were obtained from an ongoing study of children visiting the emergency department at Soroka University Medical Center (Be’er Sheva, Israel) (17). Each weekday, the first 4 Jewish children and the first 4 Bedouin children under the age of 5 years who visited the emergency department were enrolled in the study. A nasopharyngeal swab was collected and cultured, and serotype was determined using Quellung reactions, as described previously (17). For each child enrolled in the study, the number of doses of PCV7 and PCV13 received was recorded. These vaccine data were previously used to represent PCV uptake in Israel, since data from the region are within the range of the average vaccine uptake nationwide (18). We calculated uptake of PCV7/13 in each month post-PCV introduction in age bands that varied in width and the ages included. Data on IPD in adults were collected as part of a national surveillance system in Israel (19).

For our primary analyses, only data from Jewish individuals were included because of different demographic characteristics of the minority populations in southern Israel (where the carriage data were drawn from) as compared with the entire country (where the IPD data were drawn from). As a further evaluation of changes in prevalence among healthy children (rather than among children visiting the emergency department), we evaluated changes in the prevalence of PCV7-targeted serotypes among healthy children living in central Israel who were sampled as part of a series of cross-sectional surveys of nasopharyngeal colonization (20, 21). The study was approved by the Soroka University Medical Center Ethics Committee and the Sheba Medical Center Ethics Committee.

### Calculation of the “population direct effect” against colonization

The observed impacts of vaccination depend upon both the direct and indirect effects of the vaccine (22). To determine which age groups are key to determining the overall impact of vaccination, it is necessary to differentiate between the direct and indirect protection among children of different ages. In practice, interpreting the association between direct protection against carriage in different groups of children and IPD patterns in adults can be confounded by transmission between groups of children (see Web Figure 1, available at https://academic.oup.com/aje). For instance, if the vaccine predominantly disrupts carriage in toddlers and toddlers are the main source of transmission to both infants and the elderly, it can appear that declines in carriage among infants are the drivers of the declines in the elderly (Web Figure 1). However, carriage in infants in that schematic would simply be an intermediate step on the casual pathway between vaccination of toddlers and declines in disease among infants.

To avoid this issue of confounding by indirect protection, we can calculate the “population direct effect,” which provides an estimate for the overall effect of the vaccine on carriage that would be expected in the absence of indirect protection (23). The quantity is simply a function of the individual-level direct efficacy of the vaccine (as measured in a randomized controlled trial) and the proportion of the population that is vaccinated. The population direct effect would typically be calculated by estimating the proportion of persons in a particular age stratum who received 1, 2, or ≥3 doses of vaccine and multiplying this by the individual-level vaccine efficacy of 1, 2, or 3 doses against colonization due to vaccine-targeted serotypes. We modified this calculation to allow for waning of vaccine-derived protection. For a given age group (*a*) and time point (*t*),

$$\begin{array}{c} {\mathrm{Pop}\_\mathrm{Direct}\_\mathrm{Effect}}_{a,t}\\ \;=\;\Sigma [I\_\mathrm{dose}\;1_{a,t,i}\times (1-\mathrm{Risk Ratio}\left(\mathrm{RR}\right)\_\mathrm{dose}\;1\\ \;\times \;\max\;{\left(1,{\mathrm{agem}}_{i}-12\right)}^{W})+I\_\mathrm{dose}\;2_{a,t,i}\\ \;\times \;\left(1-\mathrm{RR}\_\mathrm{dose}\;2\times \;\max\;{\left(1,{\mathrm{agem}}_{i}-12\right)}^{W}\right)\\ \;+I\_\mathrm{dose}\;3_{a,t,i}\times (1-\mathrm{RR}\_\mathrm{dose}\;3\\ \;\times \;\max\;{\left(1,{\mathrm{agem}}_{i}-12\right)}^{W})]/N_{a,t}. \end{array}$$

This quantity is summed across all persons in a particular age group surveyed during a specific month. *I*_dose *K*_*a,t,i*_ is an indicator for whether a person had received 1, 2, or ≥3 doses of PCV7/13; agem_*i*_ is the age of the individual in months; *W* is the rate of waning and is set to 0.11 per month based on a meta-analysis of waning protection against carriage (24); and *N*_*a,t*_ is the number of persons surveyed in a particular month in a particular age group. We assume no waning of vaccine efficacy during the first year of life, followed by protection that wanes at a constant rate after 12 months of age, regardless of the number of doses a person has received. Estimates of vaccine efficacy (1 − RR_dose *K*) were obtained from a randomized controlled trial of PCV7 among children in Israel that used a variety of dosing schedules (14, 25). The point estimate for efficacy against colonization in the first year of life was estimated at 0%, 27%, and 46% for 1, 2, and 3 doses, respectively. To account for effects of catch-up vaccination, we made some simplifying assumptions based on previous studies (14): For children who had received 1 dose of PCV and were over the age of 12 months (i.e., ≥12 months), we assumed they had initial efficacy equal to that in an infant who had received 2 doses (27%). For children who received 2 doses of PCV and were over the age of 12 months, we assumed that these were both catch-up doses, with protection equal to that in a child who had completed the full 3-dose series (46% initial efficacy). In sensitivity analyses, we varied the rate of waning and the estimated efficacy, but these did not qualitatively alter the conclusions of the main analysis.

The observed estimates of the population direct effect for any given time point and stratum were based on small numbers, so we used cubic splines to smooth the trajectory of the population direct effect. This was accomplished using PROC GAM in SAS, version 9.4 (SAS Institute, Inc., Cary, North Carolina), where the outcome was the observed estimates of the population direct effect, and time was modeled with a cubic spline with 3 degrees of freedom. Separate smoothing models were fitted for each age range.

### Evaluating the association between direct protection and indirect effects

We fitted Poisson regression models where the outcome was the number of cases of IPD due to PCV7-targeted serotypes in a particular month and specific adult age group, and the sole covariate was Pop_Direct_Effect_*a,t*_, corresponding to a specific age group of <5 years. We controlled for seasonality using monthly dummy variables. We used only data from the Jewish population for this analysis. We compared the likelihood of each model given the data based on the Akaike Information Criterion (AIC) (26). Model likelihoods were calculated by comparing the AIC score for model *i* with the AIC score from the best-fitting (lowest AIC score) model (26):

$${\mathrm{Model}}_{i}\;\mathrm{Likelihood}=\exp(-1/2\times \left({\mathrm{AIC}}_{i}–{\mathrm{AIC}}_{\min}\right)).$$

By convention, models that were within 2 AIC points of the best model were considered to not be meaningfully different from the best model (27). To obtain a summary measure of the importance of each age group (i.e., ≤5 months, 6–11 months, 12–17 months), we averaged the likelihoods for all models in which the age group for the population direct effect variable covered that 6-month age group.

### Evaluating the association between carriage in different groups of children and IPD in adults

The relationship between carriage prevalence in children and IPD in adults can be difficult to interpret because of indirect protection (Web Figure 1). Nonetheless, carriage prevalence provides the most directly observable measure of vaccine-associated changes in transmission in children. We fitted Poisson regression models where the outcome was the number of IPD cases due to PCV7-targeted serotypes in a particular month and adult age group. The sole covariate was log(carriage prevalence) in a given age group at each time point. Log(carriage prevalence) was smoothed using PROC GAM, as described above. For these analyses, we focused on PCV7 serotypes only (rather than the 6 additional serotypes that were added to PCV13) because there was a brief period of time between the introduction of PCV7 and the introduction of PCV13, which made it difficult to disentangle early serotype replacement from vaccine-associated effects. Since the PCV7 serotypes are present in both vaccines, focusing on these serotypes allowed for more interpretable trajectories.

## RESULTS

### Description of the population

**Figure 1.**
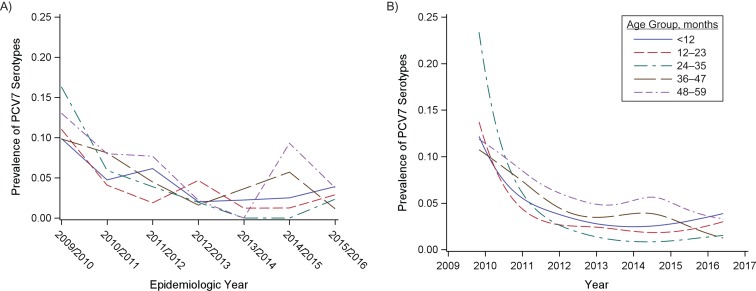
Changes in the prevalence of carriage of vaccine-targeted pneumococcal serotypes following the introduction of vaccine, Israel, 2009–2016. A) Observed proportion of nasopharyngeal swabs that were positive for 7-valent pneumococcal conjugate vaccine (PCV7) serotypes among Jewish children under 5 years of age (in 1-year age groups), by epidemiologic year (July to June). B) Smoothed proportion of children carrying PCV7 serotypes in each month during the period 2009–2016.

**Figure 2.**
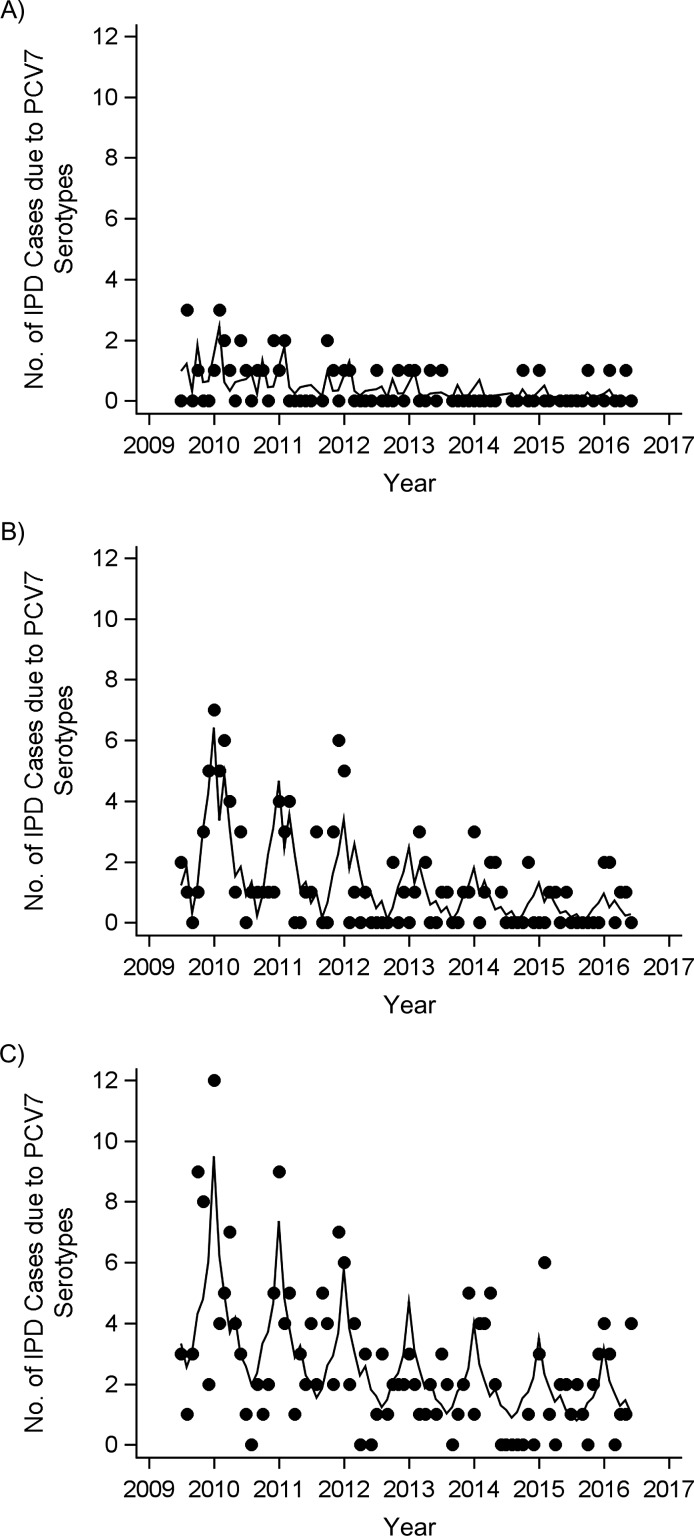
Numbers of cases of invasive pneumococcal disease (IPD) due to 7-valent pneumococcal conjugate vaccine (PCV7) serotypes among Jewish adults, Israel, July 2009–June 2016. A) Adults aged 18–39 years; B) adults aged 40–64 years; C) adults aged ≥65 years. The observed number of cases in each age group is indicated by dots. The black line shows the smoothed trend (fitted with a generalized additive model with a spline for time and monthly dummy variables).

Nasopharyngeal swabs and vaccine status were obtained from 4,464 Jewish children under 5 years of age who visited the emergency department between 2009 and 2016. Among these children, the prevalence of PCV7 serotypes declined from 21% in 2009 to 2.6% in 2016. These declines were apparent in all age groups of children under 5 years of age, with the most rapid initial declines being observed among children aged 12–23 months and 24–35 months and slower declines being observed among children aged <12 months and 36–59 months (Figure 1, Web Figure 2). The differences in these trajectories between age groups were similar among healthy Jewish children living in central Israel (Web Figure 3). During the same time period, the proportion of IPD cases in adults caused by vaccine-targeted serotypes declined (Figure 2). These declines coincided with a rapid increase in vaccine uptake among children.

### Estimated increase in population direct protection against carriage by age group

**Figure 3.**
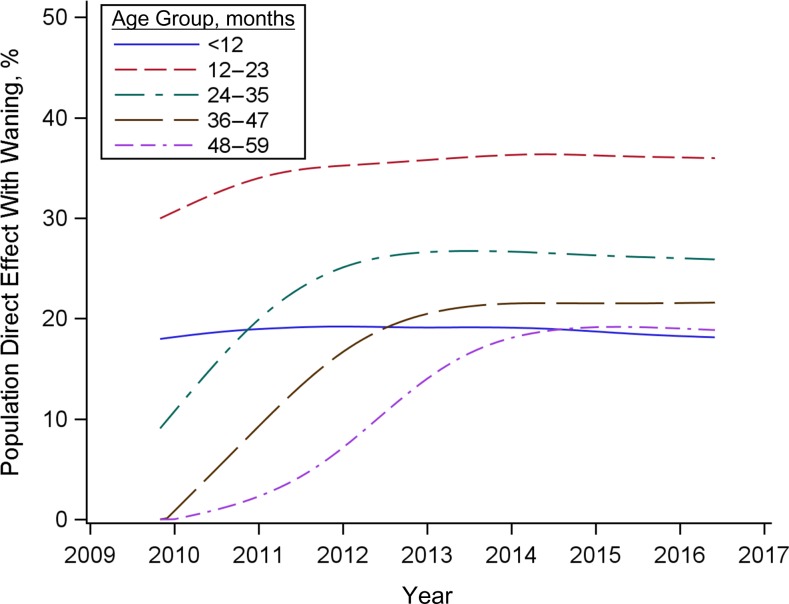
Increase in the population direct effect of 7-valent pneumococcal conjugate vaccine against pneumococcal carriage for Jewish children under 5 years of age (in 1-year age groups), Israel, 2009–2016. The population direct effect represents the vaccine effectiveness that would be expected if there were no effect of the vaccine on transmission. Estimates were smoothed with splines prior to plotting.

We estimated the reduction in the prevalence of PCV7-targeted serotypes that would be expected in the absence of an effect of the vaccine on transmission (the “population direct effect”). Among children under 12 months of age, the population direct effect reached a plateau at the beginning of the study period and remained stable at 15%–20% (Figure 3). This was because the vaccine effectiveness against carriage of the 2 primary doses received in the first year of life is low, and uptake of these 2 doses among infants was stable throughout the study period. Among children aged 12–23 months, the population direct effect against carriage increased rapidly following vaccine introduction and had plateaued at approximately 30% by 2012. Among older age groups, the increase in the population direct effect was delayed until cohorts of children vaccinated as infants aged into the specified age group (Figure 3). The maximum population direct effect achieved was lower in older age groups because of waning of vaccine-induced immunity.

### Association of an increase in direct protection against carriage with indirect protection against IPD

**Figure 4.**
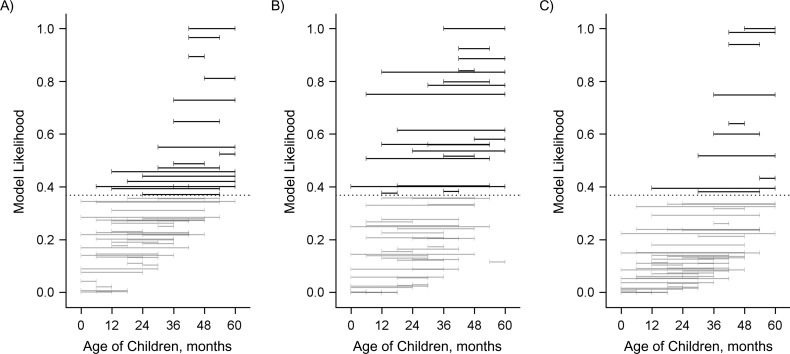
Relative goodness of fit of regression models evaluating the association between the population direct effect (PDE) of 7-valent pneumococcal conjugate vaccine in children of different age ranges (indicated by the horizontal bars) and invasive pneumococcal disease (IPD) in adults, Israel, 2009–2016. A) Adults aged 18–39 years; B) adults aged 40–64 years; C) adults aged ≥65 years. Each horizontal bar indicates a specific age range in which the PDE was calculated on the basis of uptake of the vaccine in that age group and the expected efficacy against colonization. The vertical position of the bar along the *y*-axis indicates the goodness of fit, as measured by the likelihood of the model given the data in comparison with the best-fitting model (which had a relative likelihood of 1). These values were calculated from the Akaike Information Criterion (AIC) score. The PDE in age ranges that are placed higher on the *y*-axis fit the adult IPD data better. Black bars above the dotted line were not meaningfully different from the best-fit model (AIC score within 2 points).

**Figure 5.**
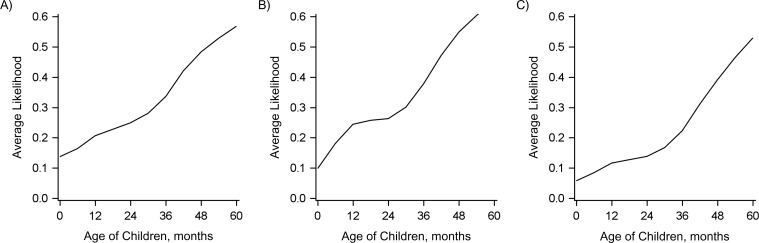
Relative goodness of fit of regression models evaluating the association between the population direct effect of 7-valent pneumococcal conjugate vaccine in children of different age ranges and invasive pneumococcal disease in adults, Israel, 2009–2016. A) Adults aged 18–39 years; B) adults aged 40–64 years; C) adults aged ≥65 years. The goodness-of-fit (model likelihood) values were averaged across all of the models in which the population direct effect included the indicated pediatric age.

We evaluated the association between the decline in the occurrence of IPD in adults and the population direct effect against carriage in different age groups of children under 5 years of age. In Figure 4, each horizontal band indicates an age range in which the population direct effect was calculated. Horizontal bands that are higher on the *y*-axis indicate a better fit to the decline in IPD due to PCV7 serotypes in adults. Bands highlighted in color and above the dashed line had strong statistical support (a difference in AIC score from the best-fitting model of ≤2). This demonstrates that the decline in IPD due to PCV7 serotypes among adults aged 18–39 years, aged 40–64 years, and aged ≥65 years was most strongly associated with the increase in the population direct effect among children aged 36–54 months, 30–59 months, and 30–54 months, respectively. However, several age groups covering the age range 6–59 months had strong statistical support. It is notable that when the age groups included children under 12 months of age, the association was significantly weaker. When all combinations of age groups were considered in aggregate (Figure 5), there was strong evidence that the direct protection among older children (i.e., ages ≥36 months) was important to explaining the decline in IPD among adults, as well as strong evidence that direct protection among children under age 12 months was not as important; the role of direct protection among children aged 12–35 months was ambiguous.

### Association of declines in carriage among infants and older children with declines in disease among adults

**Figure 6.**
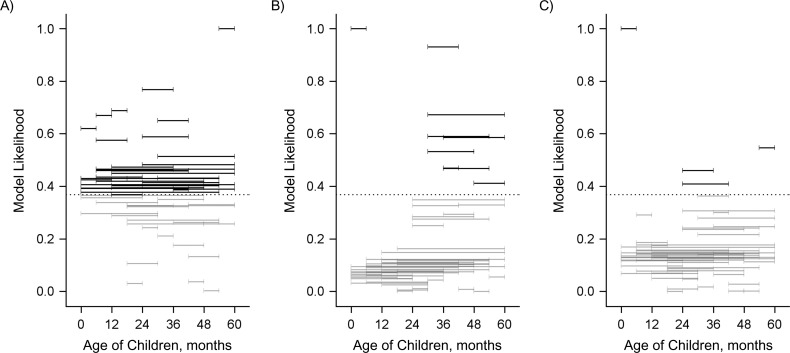
Relative goodness of fit of regression models evaluating the association between the prevalence of 7-valent pneumococcal conjugate vaccine serotypes among healthy children in different age ranges (indicated by the horizontal bars) and invasive pneumococcal disease (IPD) in adults, Israel, 2009–2016. A) Adults aged 18–39 years; B) adults aged 40–64 years; C) adults aged ≥65 years. Each horizontal bar indicates an age range in which (smoothed) carriage prevalence was calculated. The vertical position of the bar along the *y*-axis indicates the goodness of fit, as measured by the likelihood of the model given the data in comparison with the best-fitting model (which had a relative likelihood of 1). These values were calculated from the Akaike Information Criterion (AIC) scores. Carriage prevalence in age ranges that are placed higher on the *y*-axis fit the adult IPD data better. Black bars above the dotted line were not meaningfully different from the best-fit model (AIC score within 2 points).

**Figure 7.**
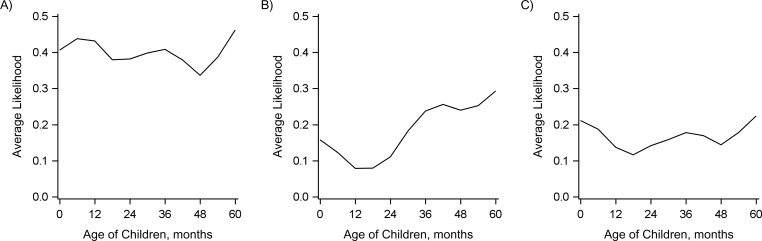
Relative goodness of fit of regression models evaluating the association between the prevalence of 7-valent pneumococcal conjugate vaccine serotypes among healthy children in different age ranges and invasive pneumococcal disease in adults, Israel, 2009–2016. A) Adults aged 18–39 years; B) adults aged 40–64 years; C) adults aged ≥65 years. The goodness-of-fit (model likelihood) values were averaged across all of the models in which the population direct effect included the indicated pediatric age.

We would expect that increasing vaccine-induced (direct) protection would cause declines in IPD in adults by reducing carriage in children. Therefore, we also evaluated the association between the decline in IPD due to PCV7-targeted serotypes in adults and the carriage prevalence of PCV7-targeted serotypes at each time point in different age groups of children. Declines in carriage of PCV7-targeted serotypes in older children (≥24 months) and children under 6 months of age were most strongly associated with the declines in IPD due to PCV7-targeted serotypes in adults (Figures 6 and 7). However, there was variability between the 3 adult age groups (18–39 years, 40–64 years, and ≥65 years) in terms of which age groups of children were most strongly correlated. These patterns could be due to direct or indirect protection in these age groups (Web Figure 1) and should therefore be interpreted with caution. Directly linking carriage prevalence with the population direct effect, the decline in carriage prevalence among children under 6 months of age was most strongly correlated with increasing population direct protection in children aged ≥12 months (Web Figure 4).

## DISCUSSION

Indirect protection has been a critical component of the overall impact of PCVs, as evidenced by the large declines in IPD among older adults who were not recipients of the vaccine. The patterns of association described here between vaccine-induced direct protection, carriage prevalence in children, and IPD patterns in adults suggest a mechanism for indirect protection. In particular, the analysis of population direct effects suggests that increasing vaccine-induced protection in toddlers and preschool-aged children drives down carriage prevalence in these older children and among children under 6 months of age. These declines in carriage among children then drive declines in IPD in adults. Therefore, direct protection of toddlers and preschool-aged children is the main causal driver of declines in IPD in adults, and carriage in infants under 6 months of age is either an intermediate step on the causal pathway or due to confounding, but it is probably not the main causal driver (Web Figure 1). Such a mechanism is consistent with the delays in indirect protection observed in adults in several high-income populations following the introduction of PCV (16, 28).

These findings have important implications for the possible success of reduced-dose vaccination schedules. Our findings suggest that vaccine uptake in infants plays a minor role in influencing the indirect effects of PCVs. This is partially because the 2 primary doses of the vaccine received in the first year of life have a weak effect on colonization. Rather, it is the third dose that provides strong protection against carriage and subsequent transmission. This suggests that if the booster dose in a 1 + 1 schedule provides adequate protection against colonization and if rates of vaccine uptake in toddlers and older children are sufficiently high, then the reduced-dose schedule should be effective at maintaining indirect protection in populations resembling the one in our study. Recent immunological data suggest that the 1 + 1 and 2 + 1 schedules provide comparable levels of immunity after the booster dose, as measured by serum immunoglobulin G levels (8). If these serum immunoglobulin G levels correlate with mucosal immunity in the nasopharynx, the reduced-dose schedule would be expected to provide effective protection against colonization that is comparable to that of a 2 + 1 schedule in these older children. These findings also suggest that catch-up campaigns targeting children aged 1–5 years could help to accelerate the realization of the indirect benefits of PCVs. Additional analyses of the timing of declines in IPD in settings that did or did not use catch-up vaccination could help in further evaluating this issue.

Our results are consistent with previous epidemiologic and modeling studies of pneumococcal colonization which suggest that toddlers and young school-aged children, rather than infants, drive transmission of pneumococcus in the population (12–15). A study of the impact of PCV7 in the United States demonstrated strong declines in IPD among children aged <12 months and 12–23 months immediately after introduction of the vaccine during the 2000 calendar year and declines in children aged 24–35 months, adults aged 40–64 years, and adults aged ≥65 years during the 2001 calendar year (28). Likewise, previous analyses demonstrated that the decline in IPD among adults in the United States was delayed by approximately 1 year in comparison with the decline in IPD among children under 5 years of age (16). These patterns reinforce the notion that it is important to maintain strong immunity to colonization in these older children in order to maintain indirect protection of younger children and adults.

Our analyses focused primarily on the Jewish population in Israel, which has a population structure and sociodemographic profile similar to that of populations in high-income countries in Europe and North America. The contact structure, age-specific prevalence, and intensity of transmission will be different in a low-income setting. As a result, the age groups that contribute most to transmission and indirect protection could differ in low-income settings as well. Performing analyses of the dynamics of timing of initiation of indirect effects in lower-income populations and in countries with or without catch-up campaigns could help in further evaluating which age groups contribute most to indirect protection.

A strength of this study was the ability to extract data on vaccine uptake and colonization at a high resolution of time and age from a large sample of children. Thus, we were able to monitor the increases in uptake, changes in carriage prevalence, and declines in vaccine-targeted serotypes at an unusually detailed level across the population. Furthermore, the IPD data were obtained from a robust national surveillance system, allowing us to quantify the indirect effects of the vaccine in adults. Limitations of the data include our reliance in the main analysis on carriage and vaccine uptake data from a study performed in an emergency department setting. However, our secondary analysis of carriage data from children living in central Israel (Web Figure 3) suggested that the trajectory of decline of PCV-targeted serotypes was similar in the general pediatric population. Finally, we used a relatively simple set of analyses based on regression models to evaluate this issue. Transmission patterns are complex and dynamic, and an age-structured transmission model could be used to further evaluate the importance of these different age cohorts in different populations and to evaluate the potential impact of changes to the vaccine schedule.

In summary, our findings suggest that direct protection of toddlers and preschool-aged children with PCVs is most influential in maintaining indirect protection against IPD among older adults in Israel. Any changes in vaccine schedules should focus on maintaining immunity in these older children.

## Supplementary Material

Web Material

## Abbreviations

AIC: Akaike Information Criterion
IPD: invasive pneumococcal disease
PCV: pneumococcal conjugate vaccine
PCV7: 7-valent pneumococcal conjugate vaccine
PCV13: 13-valent pneumococcal conjugate vaccine
