# Supplementary material for: Association Between the Decline in Pneumococcal Disease in Unimmunized Adults and Vaccine-Derived Protection Against Colonization in Toddlers and Preschool-Aged Children
Source: Am J Epidemiol. 2018 Nov 20;188(1):160–8. doi: 10.1093/aje/kwy219 (PMC6321804; doi:10.1093/aje/kwy219)

Web appendix for: **Association Between the Decline in Pneumococcal Disease in Unimmunized Adults and Vaccine-derived Protection Against Colonization in Toddlers and Preschool-aged Children**

Daniel M. Weinberger, Virginia E. Pitzer, Gili Regev-Yochay, Noga Givon-Lavi, and Ron Dagan

Web Figures 1-4

Web Figure 1

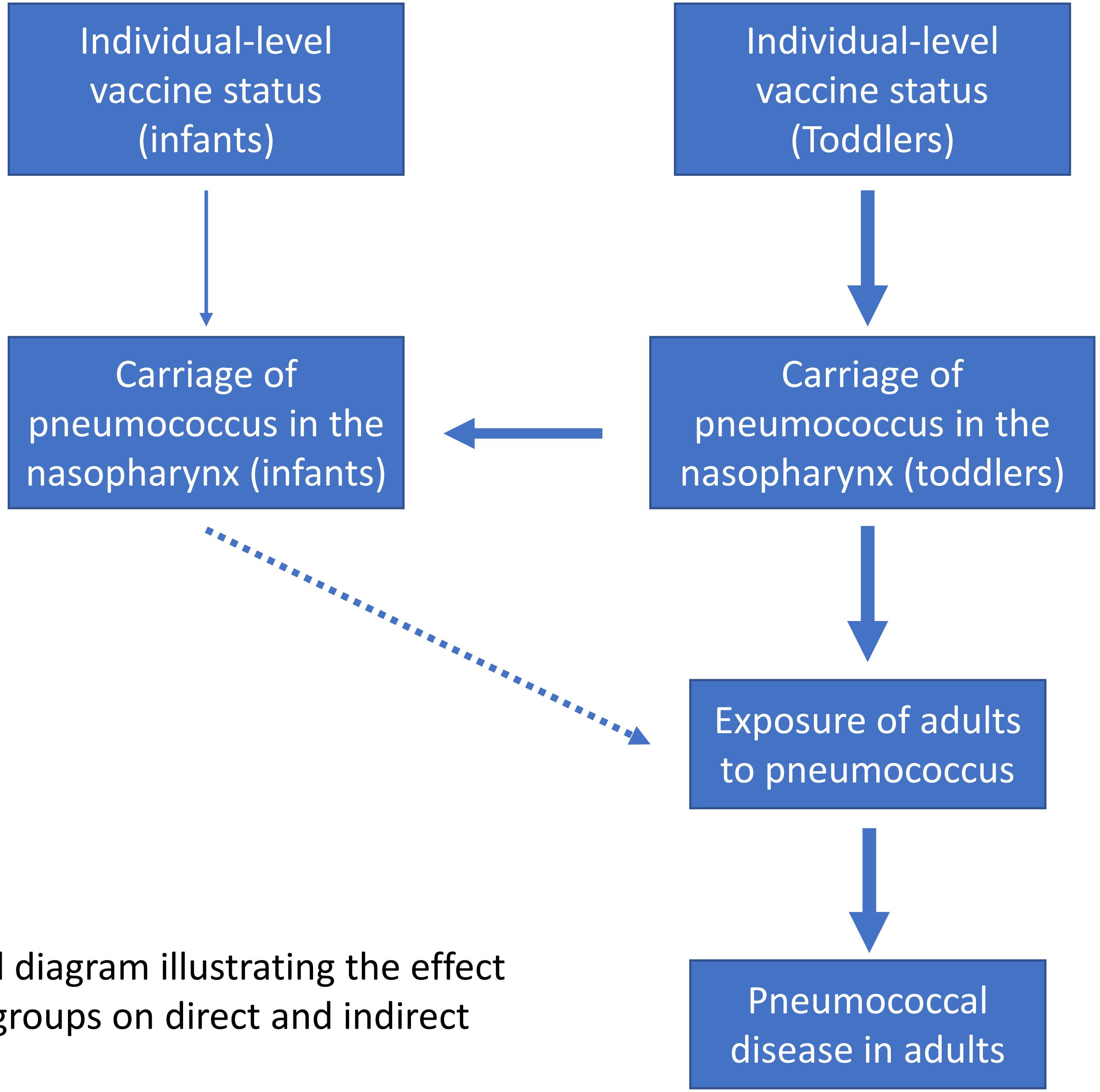

Web Figure 1: Schematic causal diagram illustrating the effect of vaccination of different age groups on direct and indirect protection.

Web Figure 2

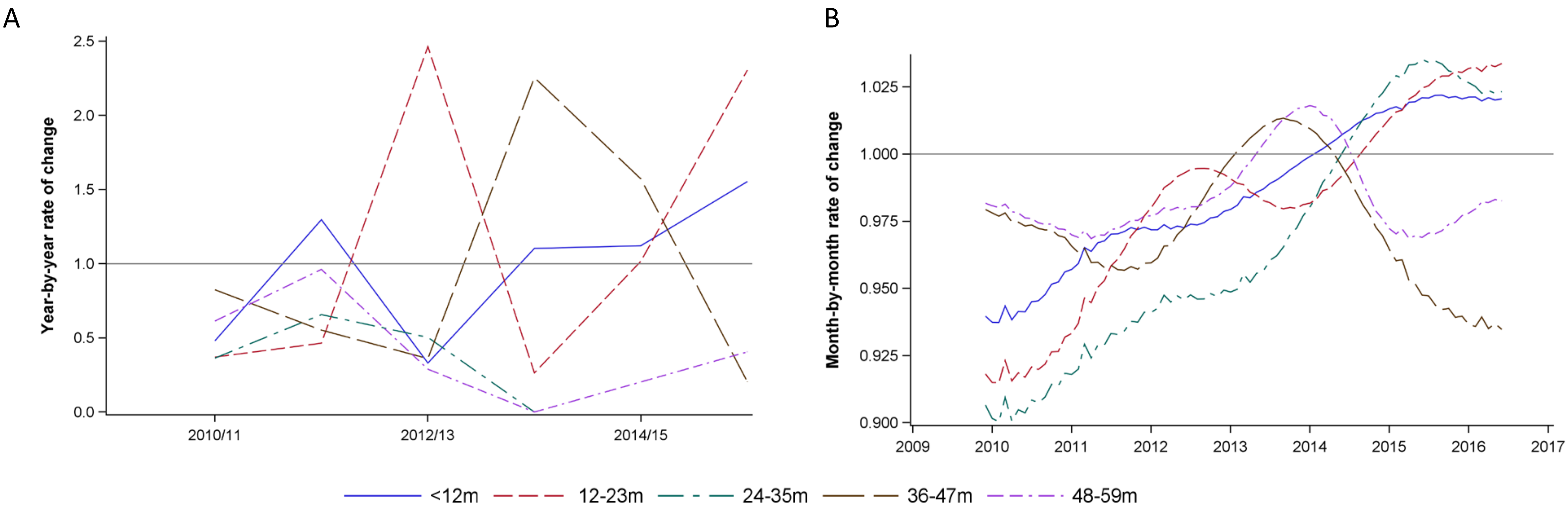

Web Figure 2: Rate of change of carriage prevalence, corresponding to those seen in Figure 1. Values further below 1 indicate faster declines. (A) The ratio of prevalence in in the indicated epidemiological year compare with the previous epidemiological year, using observed data. (B) The ratio of prevalence in the indicated month vs the previous month, using smoothed prevalence curves.

Web Figure 3

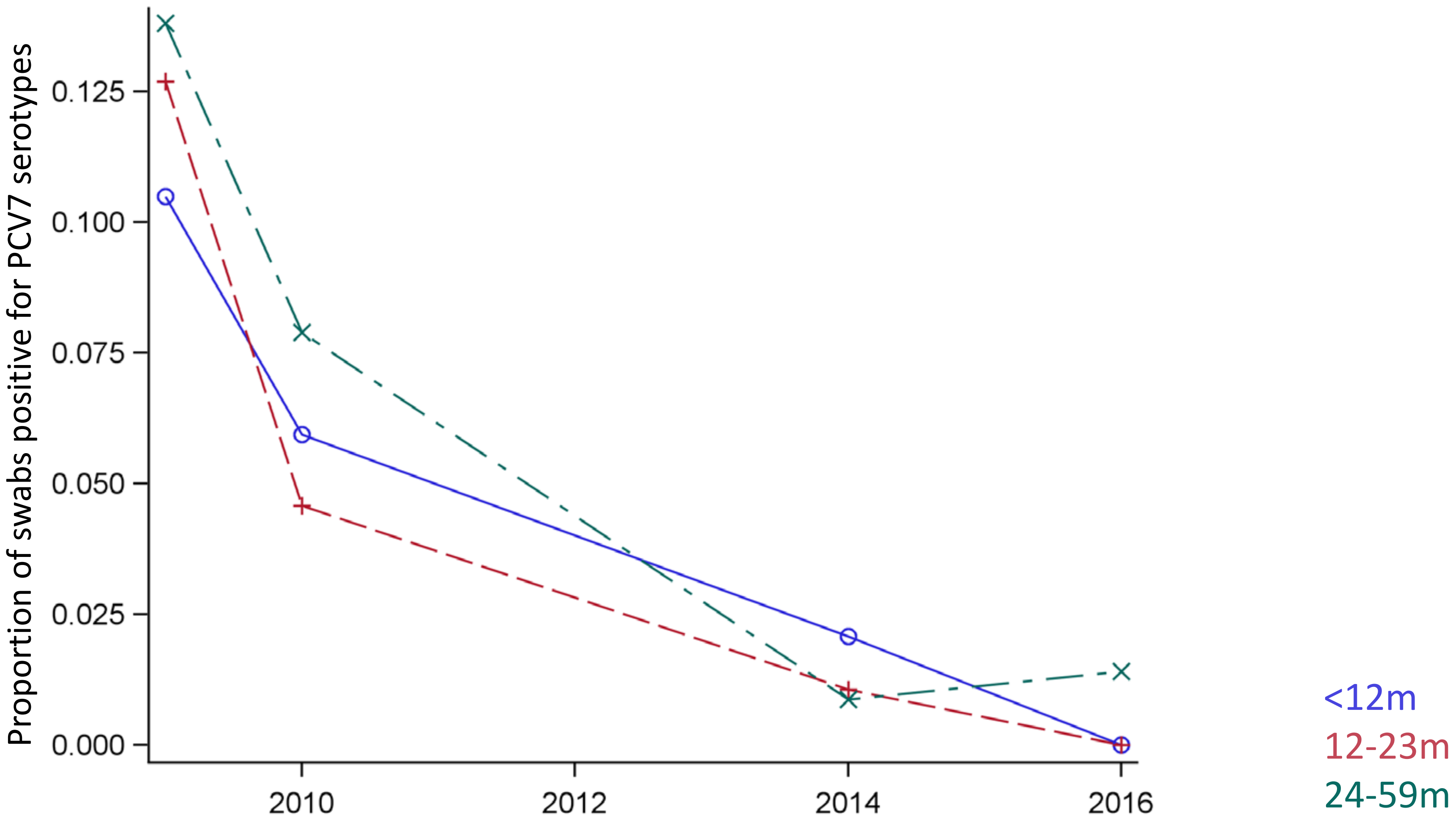

Web Figure 3: Observed proportion of nasopharyngeal swabs that were positive for PCV7 serotypes by calendar year among Jewish children <5 years of age living in central Israel, stratified into age categories (<12m, 12-23m, 24-59m).

Web Figure 4: Relative goodness of fit between the population direct effect in different age ranges of children (indicated by the horizontal bars) and the prevalence of PCV7 serotypes among children <6 months of age. Each horizontal bar indicates an age range in which the population direct effect was calculated. The vertical position of the bar along the y-axis indicates the goodness of fit, as measured by the likelihood of the model given the data compared to the best-fitting model (which has a relative likelihood of 1). These values are calculated from the Akaike Information Criteria scores. Population direct effect estimates for age ranges that are placed higher on the y-axis fit the adult IPD data better. Bars colored in red were not meaningfully different than the best-fit model (AIC score within 2 points). Some bars had near-equivalent livelihoods and overlapping ranges, each age range only has hash marks at the end of the bar. The bottom panel represents the average goodness of fit at each age.

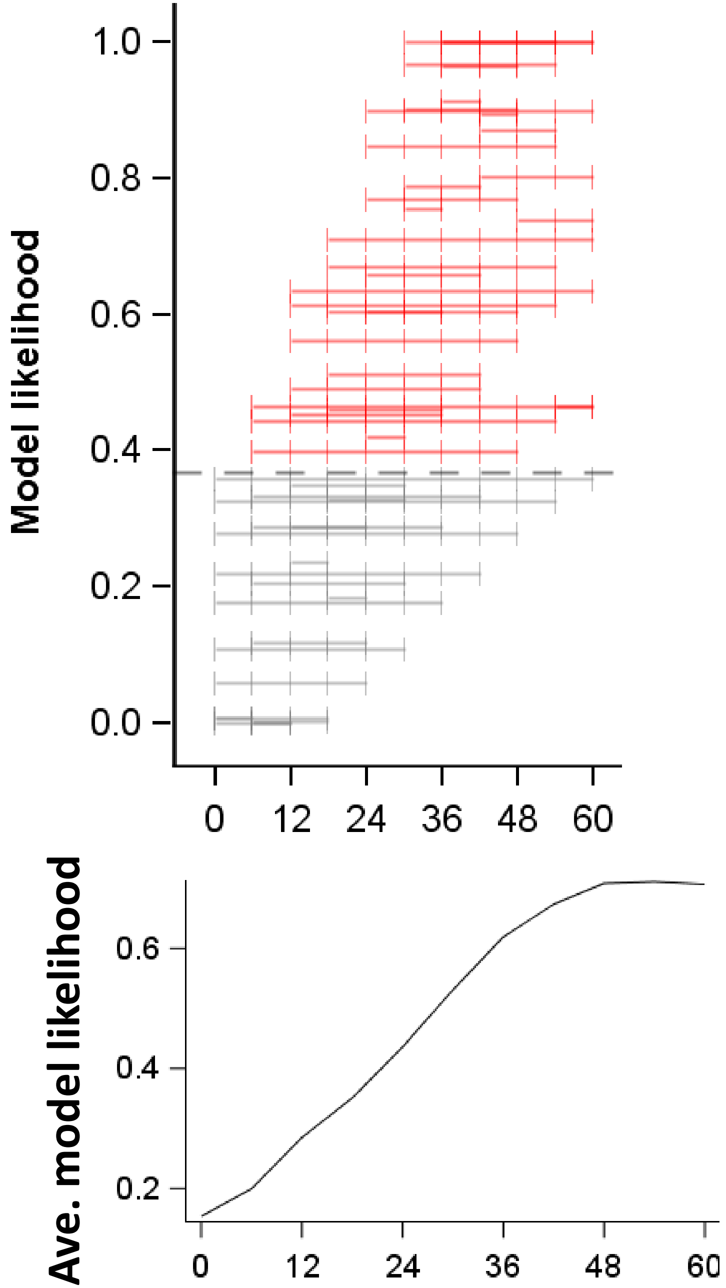

Supplement: Web Material [file kwy219_weinberger_web_material_final.pdf]
